# Various autogenous fresh demineralized tooth forms for alveolar socket preservation in anterior tooth extraction sites: a series of 4 cases

**DOI:** 10.1186/s40902-015-0026-0

**Published:** 2015-09-03

**Authors:** Eun-Suk Kim, In-Kyung Lee, Ji-Yeon Kang, Eun-Young Lee

**Affiliations:** 1 Department of Oral and Maxillofacial Surgery, Weerae Dental Clinics, Seoul, South Korea; 2 Department of Periodontology, Jukjeon Dental Hospital, Dankook University College of Dentistry, Yongin, Korea; 3 Department of Oral and Maxillofacial Surgery, Dongtan Sacred Heart Hospital, Hallym University, Hwaseong, Korea; 4 Deptartment of Oral and Maxillofacial Surgery, Chungbuk National University College of Medicine and Medical Research Institute, 52 Naesudong-ro, Heungduk-Gu, Cheongju, Chungbuk 361-763 Korea

**Keywords:** Alveolar socket preservation, Bone regeneration, Biocompatible, Bone substitute, Autogenous demineralized tooth

## Abstract

The aim of this study was to evaluate the clinical relevance of autogenous fresh demineralized tooth (Auto-FDT) prepared at chairside immediately after extraction for socket preservation. Teeth were processed to graft materials in block, chip, or powder types immediately after extraction. Extraction sockets were filled with these materials and dental implants were installed immediately or after a delay. A panoramic radiograph and a conebeam CT were taken. In two cases, tissue samples were taken for histologic examination.

Vertical and horizontal maintenance of alveolar sockets showed some variance depending on the Auto-FDT and barrier membrane types used. Radiographs showed good bony healing. Histologic sections showed that it guided good new bone formation and resorption pattern of the Auto-FDT.

This case series shows that Auto-FDT prepared at chairside could be a good material for the preservation of extraction sockets. This study will suggest the possibility of recycling autogenous tooth after immediate extraction.

## Background

Various methods and graft materials have been used to maintain alveolar ridge dimensions after tooth extraction, and these methods focus mainly on the preservation of hard tissue [1, 2]. Recently, the use of tooth grafts has increased in alveolar bone defects [3, 4]. Demineralized teeth can provide effective graft material [5]. However, immediate bone grafting after extraction used to be impossible because conventional decalcification takes three to five days. We adopted an optimized ultrasonic technology with periodic negative pressure and temperature control to enable the chairside preparation of tooth graft material. This approach dramatically reduced preparation time to ≤ 120 min (block or chip type) and 40 min (powder type), while aseptic conditions were maintained in an isolated individual bioreactor tube system, this ability to prepare block or powder increases clinical availability [4]. Most importantly, tooth extraction and grafting for socket preservation can be performed on the same day [4].

**Table 1:** Summary of patients who underwent socket preservation using Auto-FDT

No.	Age & Gender	Extracted tooth number	Defect of alveolar bone	Sites of SP	Type of Auto-FDT	Membrane	Implant sites & sizes (mm)	Delayed implant OP
1	74/F	31, 32, 41, 42	Vertical & horizontal	31, 32, 41, 42	Block	No	32, 42: 3.5 × 13	Delayed: 3 months
2	43/M	11, 21	Vertical & horizontal with labial plate destruction	11, 21	Block	Biosorb™: resorbable	11: 4.0 × 11.5, 12: 3.5 × 13	Delayed: 4 months
3	57/M	32, 38	Vertical & horizontal with lingual plate destruction	31, 32	Chip	CTi-mem™: non-resorbable	33, 42: 3.5 × 13	Simultaneous with SP
4	57/M	11, 12, 21, 22 31, 32, 41, 42	Vertical & horizontal without labial plate destruction	11, 12, 21, 22, 31, 32, 41, 42	Powder	Colla tape: resorbable	12, 22: 4.0 × 11.5, 32, 42: 3.5 × 13	Simultaneous with SP

*SP* socket preservation, *Auto-FDT* autogenous fresh demineralized tooth graft, *OP* operation

The aim of this case report was to present the clinical usefulness of socket preservation using autogenous fresh demineralized tooth (Auto-FDT) prepared at chairside in anterior teeth extraction sites.

## Case presentation

This study was approved by the Dankook University Jukjeon Dental Hospital institutional review board (2012–004) and all participants signed an informed consent agreement (Table 1).

### Case 1

**Fig. 1:**
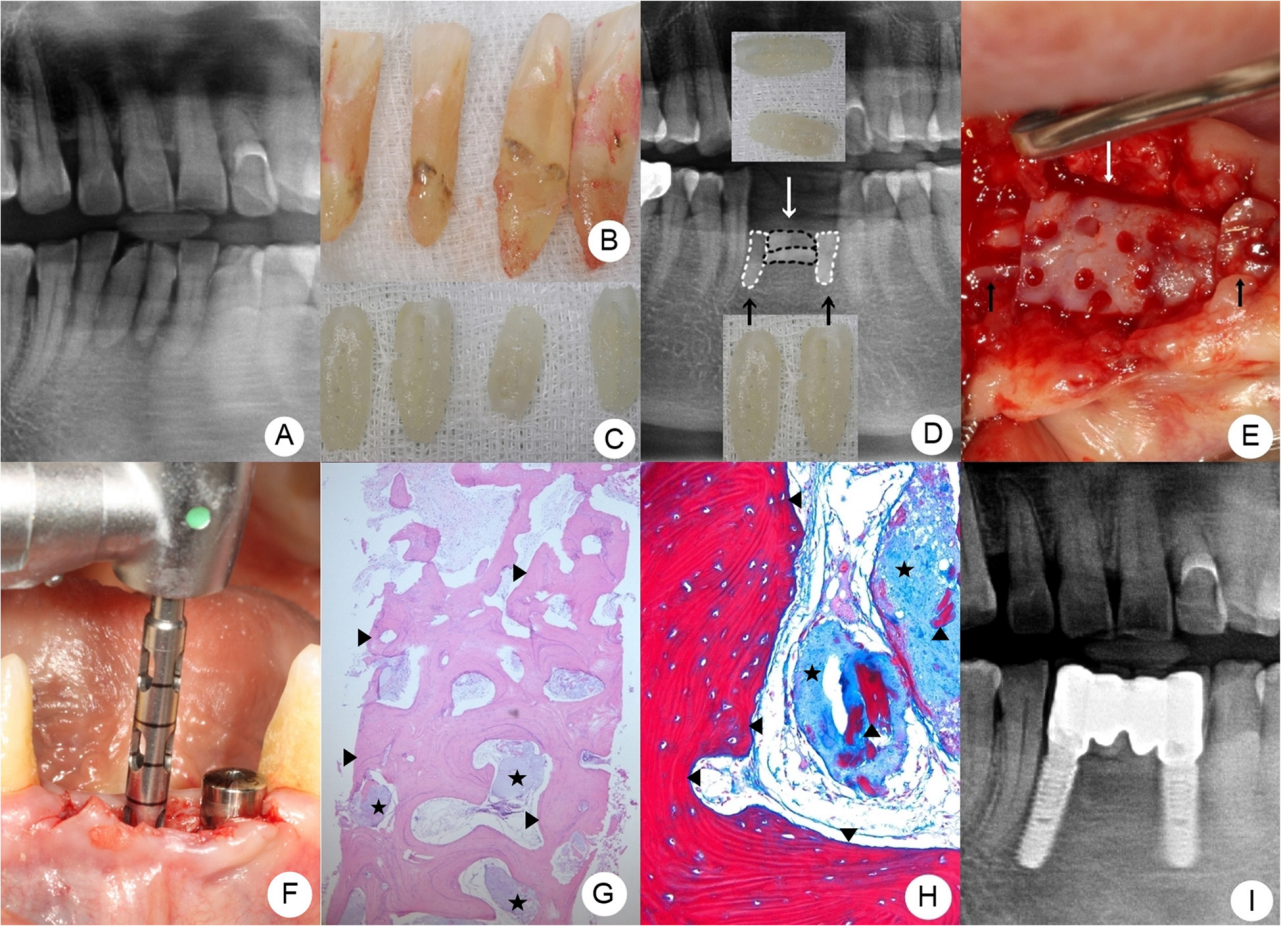
Clinical and radiographic images of case 1. **a** Panoramic view before extraction of # 31, 32, 41, 42. **b** Extracted lower anterior teeth. **c** Auto-FDT graft material (block type). **d**, **e** Blocks of Auto-FDT were inserted into the extraction socket vertically (black arrow) or horizontally (white arrow) depending on defect shape. **f** Bone core was taken at 3 months after the socket preservation. **g** Histologic section at postoperative 3 months showing that the Auto-FDT was almost completely replaced by new bone (H&E, x40). **h** Higher magnification showing new bone in resorbable Auto-FDT (MT, x200). Auto-FDT (asterisk), new bone (black arrow head). **i** Panoramic view at 18 months after socket preservation surgery

Four anterior teeth (#32-42) showed advanced periodontitis with mobility and severe alveolar resorption in a 74-year-old female (Fig. 1a). The extracted teeth were converted into block type Auto-FDT (Fig. 1b-e). No barrier membrane was used. After 3 months, graft sites were surgically reentered for implant placement. Socket preservation sites had maintained good, satisfactory bone and soft tissue contours for implant surgery despite slight horizontal resorption. Dental implants (TSIII CA, Osstem, Seoul, Korea) were placed in #32, 42 sites and achieved initial stability with an insertion torque 20–30 Ncm. At 3 months after implant placement, 4-unit fixed prosthesis was placed. A bone core was taken from the center of socket preservation sites and histologic sections were prepared at implant placement (Fig. 1f). It showed new bone around the grafted Auto-FDT throughout the whole specimen. A wide range in the quantity of new bone formation was noted (Fig. 1g-h). Good alveolar ridge height without bony resorption was achieved at 18 months after socket preservation surgery (Fig. 1i).

### Case 2

**Fig. 2:**
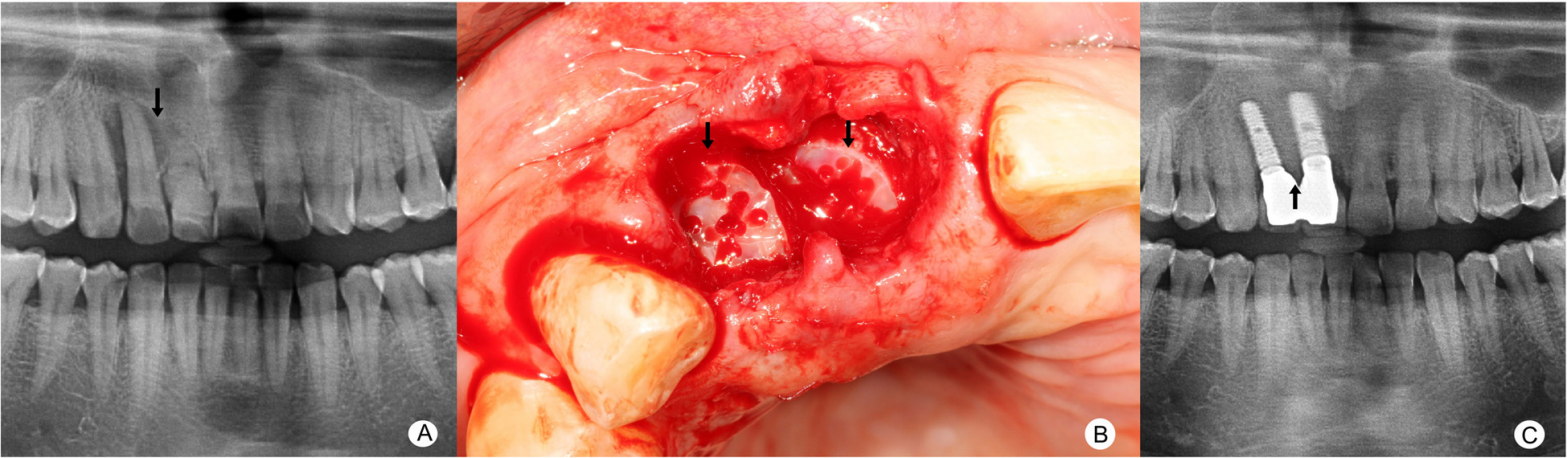
Clinical and radiographic images of case 2. **a** Panoramic view before extraction of #11, 12. A combined periodontic-endodontic lesion and root fracture of #11 were diagnosed (black arrow). **b** Blocks of Auto-FDT (root portion, black arrow) were inserted into extraction sockets. **c** At 26 months after socket preservation, regenerated bone showed good maintenance between implants in panoramic view (black arrow)

A panoramic radiograph of a 43-year-old male revealed root fracture of #11 and alveolar bone destruction around #11, 21 (Fig. 2a). The blocks of Auto-FDT and collagen membrane (Biosorb™, 3 M ESPE, USA) were used to fill sockets for implant placement after wound healing (Fig. 2b). At four months after socket preservation with block Auto-FDT, the graft was well consolidated but the amount of healed bone was slightly less than their initial quantities. Two implants were placed in #11, 12 areas. At 4 months after implants placement, final restorations were completed. The patient was periodically recalled and followed up after prosthetic restoration for 19 months (Fig. 2c). No implants loss occurred, but more horizontal resorption was observed than in the other cases.

### Case 3

**Fig. 3:**
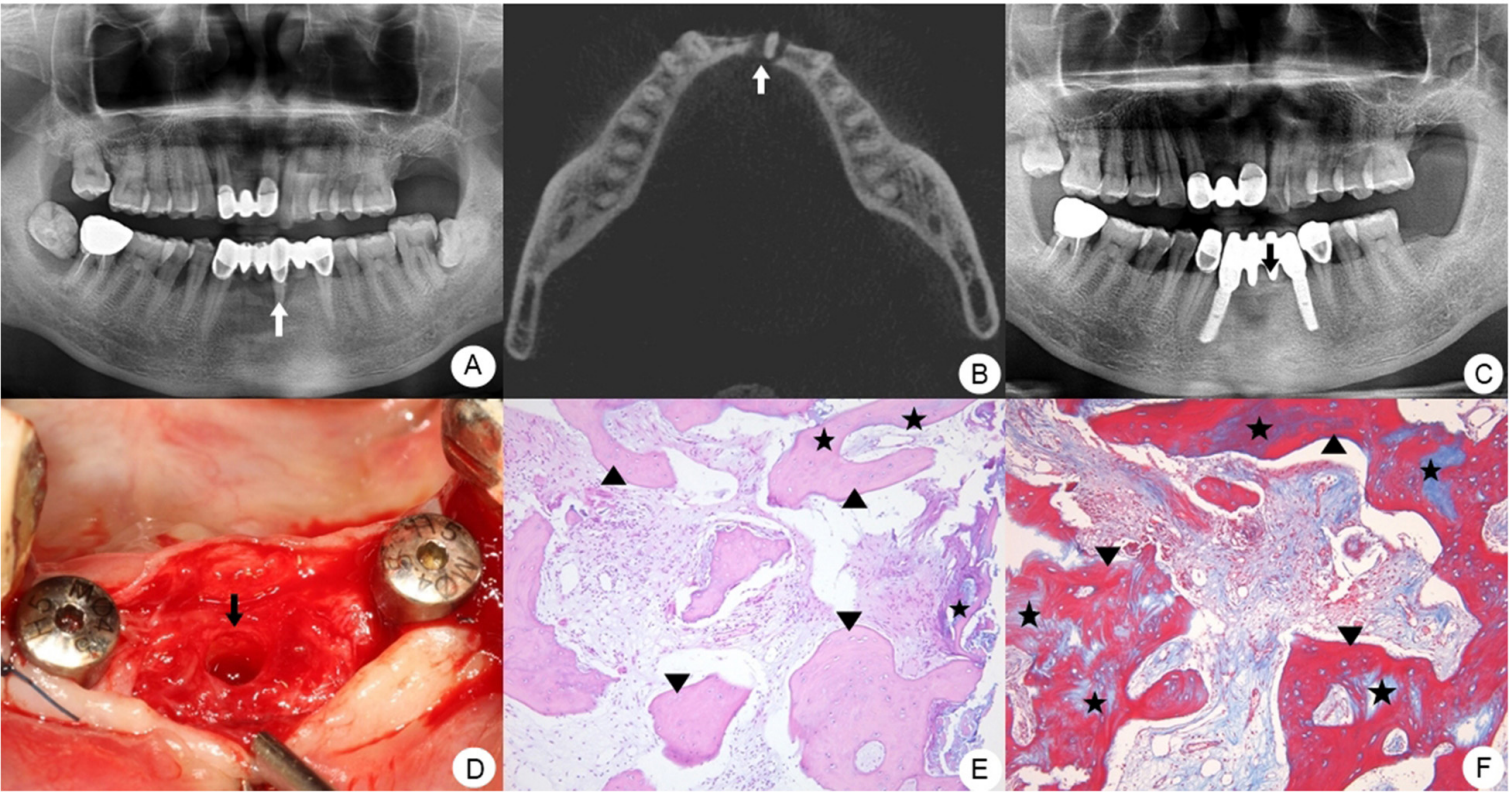
Clinical, radiographic and histologic images of case 3. **a** Preoperative panoramic view (white arrow: bony defect). **b** Preoperative conebeam CT (white arrow: bony defect). **c** Panoramic view at 33 months after socket preservation (black arrow: alveolar crest). **d** A bone biopsy site in the center of a socket preservation site (black arrow: trephine drill hole). **e** Histologic section taken at 5 months after socket preservation showing remodeling of new bone around the Auto-FDT (H&E, x100). **f** A Masson’s trichrome stained section showing integration between newly formed bone and Auto-FDT (MT, x100). Auto-FDT (asterisk), new bone (black arrow head)

A panoramic radiograph and conebeam CT of a 57-year-old male showed an alveolar bone defect around #32 including #31 edentulous sites showing vertical and horizontal resorption with lingual plate destruction on lower incisors areas (Fig. 3a and b). Teeth (#32, 38) were used to prepare chip Auto-FDT; prepared block Auto-FDT within 120 min of extraction and then was changed to chip using a bone mill.

Extraction sockets of #32 and the adjacent defect were filled with chip Auto-FDT. To prevent dissemination of particles and maintain of alveolar bone, a screw fixed thin titanium sheet (CTi-mem™, Neobiotech, Seoul, Korea). The patient was followed for 33 months after the socket preservation. The follow-up panoramic radiography showed a good alveolar ridge height without bony resorption (Fig. 3c).

A bone trephine bur of external diameter 2 mm was used to obtained a bone core from the centers of socket preservation sites at the uncovering surgery (Fig. 3d). In histologic sections, new bone (woven and lamella type) and a remnant of resorbed Auto-FDT were observed. Fibrous tissue and blood vessels were also found (Fig. 3e). In the MT stained section, the interface between resorbed Auto-FDT and new bone was tight and interconnected (Fig. 3f).

### Case 4

**Fig. 4:**
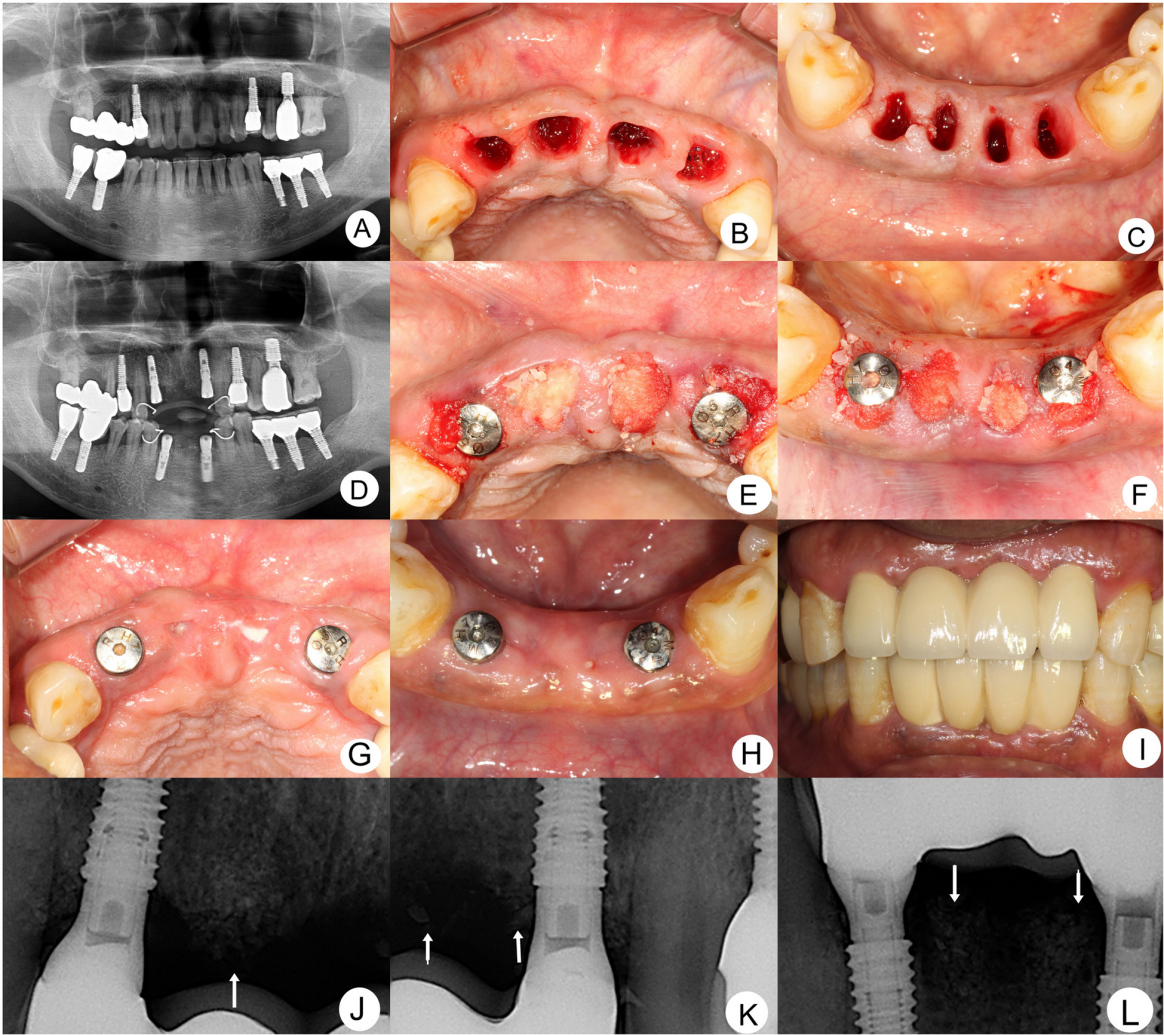
Clinical and radiographic images of case 4. **a** Horizontal and vertical alveolar bone resorption were observed on upper and lower anterior teeth in panoramic view. **b** Extracted upper incisors sites. **c** Extracted lower incisors sites. **d** Implant installation on lateral incisors and socket preservation on extraction sites in immediate post-operative panoramic view. **e** Extraction sockets on #11, 21 and labial sides of implant fixation on #12, 22 were filled with powder Auto-FDT and Colla tape. **f** Extraction sockets on #31, 41 and labial sides of implant fixation on #32, 42 were filled with powder Auto-FDT and Colla tape. **g**,**h** Epithelial closure of socket preservation sites was achieved at 2 weeks after socket preservation. **i** At 5 months after socket preservation, 4-unit fixed bridges were placed. Intro-oral photo: The final restorations were delivered. **j-l** Periapical x-ray views: At six months after final prosthesis placement, regenerated bone appeared to support the implant well. (white arrow: maintenance of the triangular bony structure on the mesial site of the implants)

A 57-year-old male’s panoramic radiograph revealed overall alveolar bone resorption (Fig. 4a). All anterior teeth (#12-22 and #32-42) were diagnosed as hopeless and extracted (Fig. 4b and c), and used to prepare powder Auto-FDT within 40 min of extraction.

We installed the implant installation (TSIII CA, Osstem, Seoul, Korea) in #12, 22, 32, and 42 with socket preservation with powder Auto-FDT (Fig. 4d). To prevent dissemination of Auto-FDT particles, a collagen sheet (Colla tape, Zimmer, Germany) covered extraction sockets (Fig. 4e and f). The healed soft tissues were observed at 2 weeks after surgery (Fig. 4g and h).

After a 4-month healing period, 4-unit fixed bridges were placed in the upper and lower sites for implant installation respectively (Fig. 4i). Clinical and radiological examinations at 6 months after prosthesis placement showed horizontal and vertical volumes of extraction sockets were well maintained (Fig. 4j-l).

## Discussion

Tooth extraction is invariably followed by loss of height and width of the alveolar process. During natural healing after extraction, reductions in width of between 2.6 and 4.6 mm and in height of between 0.4 and 3.9 mm are observed [6], and these result in narrowing and shortening of the residual ridge [7]. Osseous augmentation procedures for creating bone volume for dental implants often involve the use of grafting materials with or without barrier membranes [1, 2, 8].

Tooth is a potentially valuable graft material, and demineralized dentin has recently been used to reconstruct alveolar bone [3, 4]. However, the long processing time required to demineralize and prepare teeth for socket preservation is problematic. Lee et al.[4] reported a new method to reduce processing times chairside, and reduced the whole preparation process of block or chip Auto-FDT to less than 120 min. It is possible to commence socket preservation immediately after extraction using the extracted tooth. Auto-FDT is an organic graft material that has been chemically treated to remove its inorganic components. The calcium (inorganic component) concentration of block Auto-FDT is 6.5 wt.% whereas that of normal dentin is 31 wt.% at a depth of 300–600 μm from external surfaces, indicating that 79 % of calcium is removed [4]. The remaining inorganic and organic components are needed in grafted areas for bone formation by osteoinduction or osteoconduction [6]. It is possible to control levels of inorganic components by changing tooth processing times.

The results of clinical and radiologic studies performed in the present study suggest that socket dimensional changes following tooth extraction are prevented or reduced using Auto-FDT. Radiographs obtained at 33 months after socket preservations revealed maintenance of alveolar ridges in case 3. In the patients treated with block Auto-FDT with or without resorbable membrane such as case 1 and 2, a slight width reduction was observed during clinical examinations, whereas heights were maintained. The reasons for resorption were that block Auto-FDT was placed with natural root size and shape into extracted sites without overcorrection and space maintained improperly with or without resorbable membrane. It was not enough to prevent the unfavorable stress of lip movement and soft tissue contracture without stress-shielding barrier membrane.

Chip Auto-FDT with a stress-shielding barrier membrane (titanium sheet) for space maintenance (case 3), it improved ridge height and width dimensions when compared to block type with/ without resorbable membrane (case 1 and 2), and it was possible to maintain of a space for graft materials using titanium sheet to prevent unfavorable stresses.

Powder Auto-FDT and chip or block Auto-FDT with a stress-shielding barrier membrane might preserve extraction sockets better with respect to height and width than the block Auto-FDT alone as used in the present study. In case 4, power Auto-FDT valuably preserved as determined by clinical and radiologic findings without dimensional changes. Periapical radiograms showed good marginal bone response and the absence of any residual vertical bone defect (Fig. 4j-l). Furthermore, the triangular shape of bone in the mesial side of the implant was maintained. The powder type is believed to be resorbed more slowly than the other two types, as the time of demineralization is less than that of the block type, which makes it possible to maintain a space for bone formation and to prevent unfavorable stress without a titanium sheet.

Several limitations of this study should be considered. In particular, it was not a prospective study and measurements of alveolar ridge dimensions were not performed. Nevertheless, it presents good results for socket preservation using Auto-FDT with respect to implant installation and alveolar ridge maintenance.

## Conclusions

Socket preservation using powder, chip or block type Auto-FDT with a stress-shielding barrier membrane was effective in maintaining ridge heights and widths for implants. Further studies are needed to determine the effects of different types of bony defect on the functions of Auto-FDT and barrier membranes.
